# Supplementary material for: Evaluation of the Abdala Vaccine: Antibody and Cellular Response to the RBD Domain of SARS-CoV-2
Source: Vaccines (Basel). 2023 Nov 30;11(12):1787. doi: 10.3390/vaccines11121787 (PMC10748004; doi:10.3390/vaccines11121787)
Supplement: Supplementary file 1 [file vaccines-11-01787-s001.zip › Supplementary Figures.pdf]

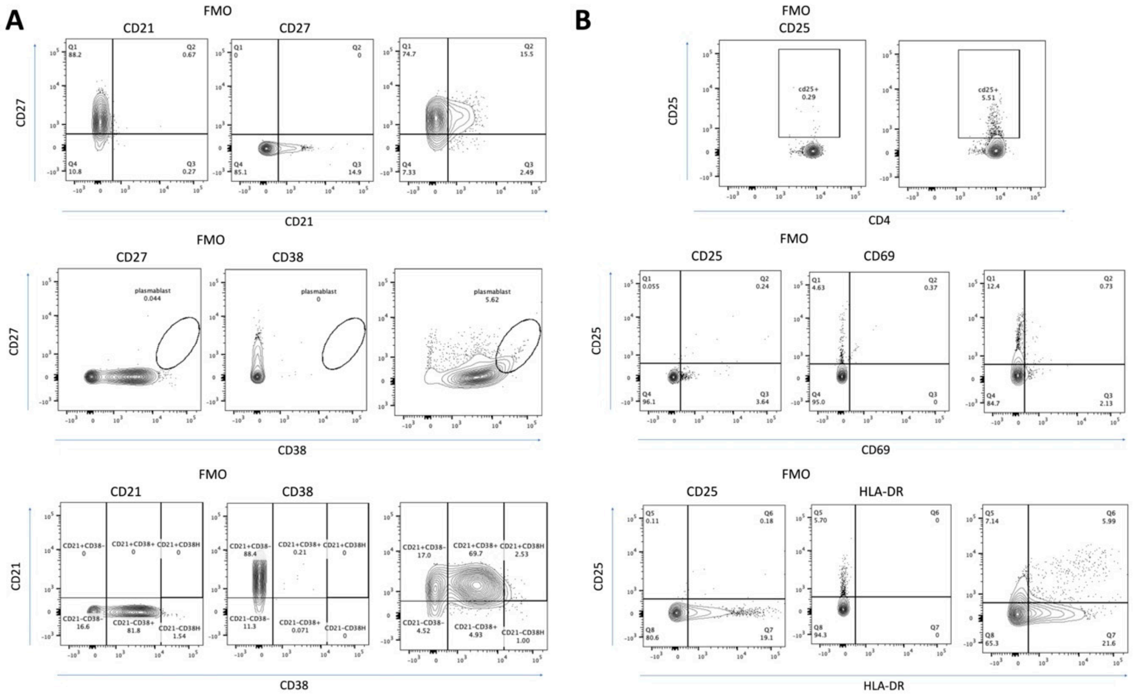

Figure S1: FMO controls. A. FMO panel for B-lymphocytes analysis, CD21 vs CD27, CD38 vs CD27, and CD38 vs CD21 dot plot from CD19+ lymphocytes. FMO for CD21, CD27 and CD38 are shown. **B.** FMO panel for T-lymphocytes analysis, CD4 vs CD25, CD69 vs CD25, and HLA-DR vs CD25 dot plot from CD3+CD4+ lymphocytes. FMO for CD25, CD69 and HLA-DR are shown.

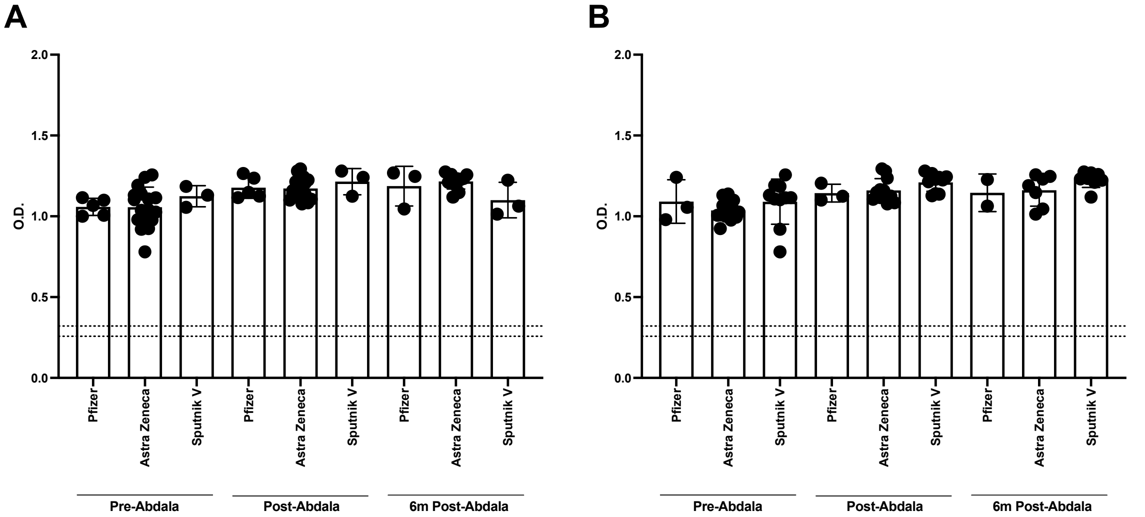

Figure S2: OD of IgGs related to SARS-CoV-2 against RDB domain of S protein. (A) OD of IgG against RBD pre- and post-Abdala vaccination and 6 months (6m) post-Abdala vaccination by initial vaccine scheme. Pre-Abdala vaccination: Pfizer n = 5, Astra Zeneca n = 17, Sputnik V n = 3; post-Abdala vaccination: Pfizer n = 5, Astra Zeneca n = 17, Sputnik V n = 3; and 6 months (6m) post-Abdala vaccination: Pfizer n = 3, Astra Zeneca n = 11, Sputnik V n = 3. **(B)** OD of IgG against RBD pre- and post-Abdala vaccination and 6 months (6m) post-Abdala vaccination by initial booster dose. Pre-Abdala vaccination: Pfizer n = 3, Astra Zeneca n = 12, Sputnik V

n = 10; post-Abdala vaccination: Pfizer n = 3, Astra Zeneca n = 12, Sputnik V n = 10; and 6 months (6m) post-Abdala vaccination: Pfizer n = 2, Astra Zeneca n = 7, Sputnik V n = 8. Mean and standard deviation (SD) are shown. Central trend values and dispersion values are indicated in Table S1 in Supplementary Data.

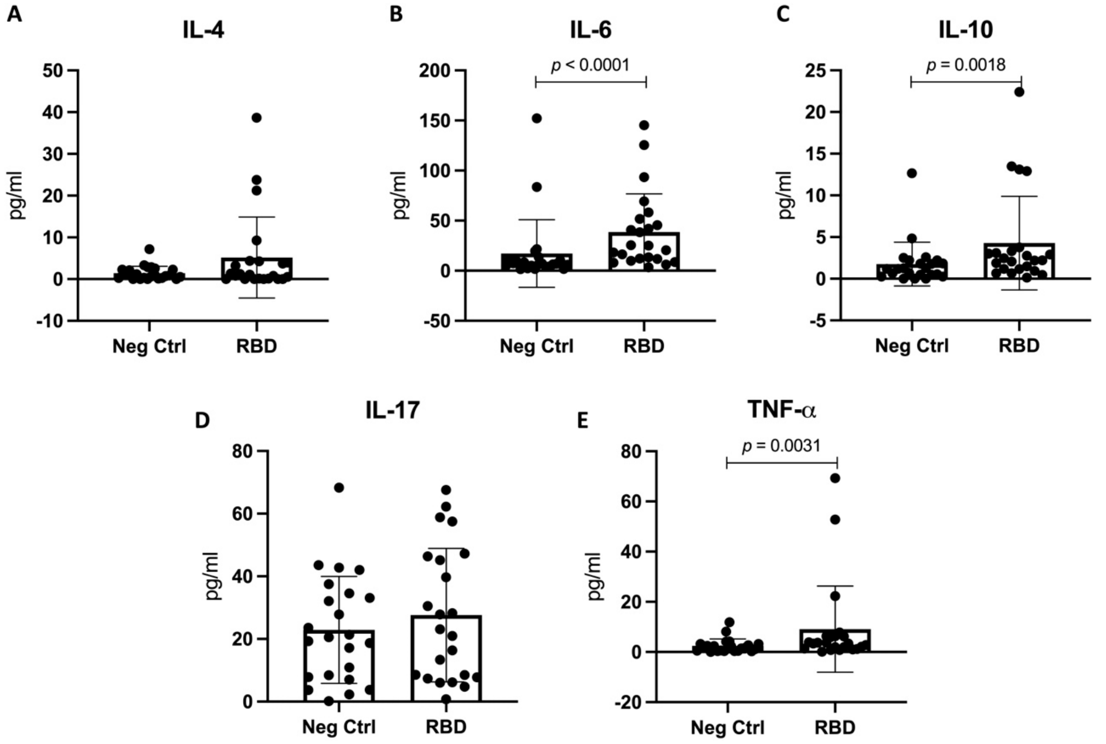

Figure S3. Concentration of cytokines after stimulus with RBD protein for 24h. A. pg/mL of IL-4. **B.** pg/mL of IL-6. **C.** pg/mL of IL-10. **D.** pg/mL of IL-17. **E.** pg/mL of TNF-α n = 23. Mean and standard deviation of mean (SD) are shown. Central trend values and dispersion values are indicated in Table S5, Supplementary Data.
